# Employee Engagement and Wellbeing in Times of COVID-19: A Proposal of the 5Cs Model

**DOI:** 10.3390/ijerph18105470

**Published:** 2021-05-20

**Authors:** María-Carmen De-la-Calle-Durán, José-Luis Rodríguez-Sánchez

**Affiliations:** Department of Business Administration (ADO), Applied Economics II and Fundaments of Economic Analysis, Universidad Rey Juan Carlos, Paseo de los Artilleros s/n, 28032 Madrid, Spain; carmen.delacalle@urjc.es

**Keywords:** employee engagement, conciliation, cultivation, confidence, compensation, communication, wellbeing, COVID-19

## Abstract

The COVID-19 pandemic has had an unprecedented impact on the labor market. The psychological pressure and uncertainty caused by the current changing workplace environment have led to negative consequences for workers. Considering the predictive relationship between employee engagement and wellbeing and in light of this unprecedented situation that affects workers of all the industries worldwide, this study aims to identify the key main drivers of employee engagement that can lead to employee wellbeing in the current context. Through a literature review, a theoretical model to strengthen engagement in times of COVID-19 is proposed. The main factors are conciliation, cultivation, confidence, compensation, and communication. Whereas prior to the pandemic, firms had already understood the need to achieve this, it is now considered a vital tool for staff health and wellbeing. This article makes two main contributions. First, it provides a model for boosting employee engagement, and therefore, wellbeing. Second, managerial suggestions are made to apply the theoretical model.

## 1. Introduction

The current pandemic situation has created a challenging context for organizations and particularly for human resource management. According to [1], there are radical changes occurring in the work and social environment, such as shifting to remote work and applying new workplace policies and procedures to limit contact. All this has led to consequences for workers, such as difficulties in disconnecting from work demands, separating work and private life, and even other psychosocial risks, such as isolation [2,3].

Essential workers have faced stressful situations, such as increased workloads, longer working hours, and reduced rest periods. In addition, an important concern is getting infected at work and passing the virus to family and others [3]. Finally, layoffs, pay cuts, and furloughs have led to increased levels of job insecurity and economic loss, resulting in increased levels of uncertainty among workers [1,3]. All this pressure at work represents an important threat to employee wellbeing [4].

In this context, companies must cope with new health protocols and measures to protect both physical and mental health [5,6]. Thus, it could be said that handling workforce issues turns out to be more challenging than before the pandemic.

Existing management research offers insights on strategies for managing human resources in these crises, such as putting people first, attending to teamwork and communication, adopting clear and humble leadership [7], providing training sessions, incentives to motivate employees, help them overcome their uncertainty, and ensure teleworking tools [8]. All these measures are aimed not only at improving wellbeing but also at recovering and improving the company’s performance, mainly through staff engagement. The study of [9] confirmed the predictive relationship between employee engagement and wellbeing. In this regard, it is also demonstrated that employee engagement and wellbeing have a positive impact on efficiency, productivity, and organizational performance [10,11,12].

Employee engagement is “a positive, fulfilling, work-related state of mind that is characterized by vigor, dedication, and absorption. Rather than a momentary and specific state, engagement refers to a more persistent and pervasive affective–cognitive state that is not focused on any particular object, event, individual, or behavior” [13,14].

The health crisis caused by COVID-19 is creating considerable uncertainty among workers. Moreover, many organizations started de-prioritizing employees’ psychological needs owing to serve business losses due to lockdown [15], which is compromising their employee engagement and wellbeing. However, the achievement of both the organizational agility and the resilience required for corporate health in times of the pandemic requires employees to be both skillful and resilient, whereby their engagement is essential [15,16].

Aside from [15] recent study, little is known about how to address engagement to achieve wellbeing in the current pandemic environment. While [15] provided a general overview of what an engagement environment should be, this article focuses on specific areas that companies can act on to achieve employee engagement. Thus, in light of this unprecedented situation that affects workers of all the industries worldwide, this study examines the main drivers of employee engagement under the current health context. Among the main research questions are:

– RQ1. What are the main factors influencing employee engagement?
– RQ2. Why are these factors relevant for wellbeing in the context of the COVID-19 health crisis?
– RQ3. How can firms address these factors to increase employee engagement in times of COVID-19?

To answer these questions, a literature review has been developed to identify and better understand how to manage these factors. Thus, by considering the psychological pressure and uncertainty caused by a changing workplace environment, such as the current one [17], our research proposes a model to strengthen employee engagement in times of COVID-19 based on five key drivers: conciliation, cultivation, confidence, compensation, and communication. Whereas prior to the pandemic, firms had already understood the need to achieve this, it is now considered a vital tool for staff health and wellbeing.

This article makes two main contributions. First, it provides a model for boosting employee engagement. Second, a roadmap has been drawn up that may serve as a reference for the different managers involved. The article is organized as follows: the first section develops the theoretical underpinnings of the factors influencing employee engagement, establishing the most pertinent organizational health and wellbeing policies. The second section presents the results obtained and analyzed, along with the direct impact expected in the crisis caused by the COVID-19 pandemic. The third section addresses the results obtained and analyzed. Finally, the conclusions and future lines of research are presented.

## 2. Methodology

To analyze the literature about employee engagement and wellbeing, a systematic review considering previously published studies about the topic was done. A systematic review of academic writing is a selective and critical examination that seeks to analyze and integrate essential information from primary research studies on a particular subject. This procedure identifies relevant studies, assesses their quality, and summarizes their results. Reviews should be done objectively, rigorously, and meticulously [18]. A systematic review requires an information search strategy based on bibliographic databases, an appropriate assessment of the studies that will be included in the subsequent analysis, and, finally, an adequate summary of the information collected [19]. The collection of information should be comprehensive in order to avoid selection bias. For this purpose, the search should be made in as many sources as possible with appropriate selection criteria [20].

This method ensures that the synthesis of the literature is made in a rigorous, transparent, and reproducible manner [21,22]. In order to choose the most suitable articles for the study, a search process was carried out, enabling us (1) to identify existing studies, (2) to analyze their usefulness and relevance in a specific research topic, and (3) to gather several studies conducted independently, at times with opposite results, and synthesize their implications [23].

The information search strategy was carried out using the bibliographic database Web of Science (WoS). This database is the most prestigious in the field of social sciences, especially in business and economics.

For the systematic review, we followed a keyword search of the literature. With this method, the researchers’ subjectivity in data collection was excluded. The research was limited to the articles and reviews published in peer-reviewed journals included in the WoS database. The search terms selected for the search were “employee engagement” AND “wellbeing”. The search took into consideration papers published from 1900 to March 2021. The document search was carried out on 06 March 2021, according to the protocol of Table 1.

The number of documents obtained was 148. All the results obtained in the search were double-checked to eliminate duplicates, to confirm they fit the subject matter of the study, and those with no identified authors were discarded. To avoid subjective decisions, three authors performed this second screening of literature. From this screening, a total of 59 articles were identified.

## 3. Theoretical Framework: Employee Engagement and Wellbeing in Times of COVID-19

Healthy organizations are supposed to introduce workplace measures and processes to promote and maintain a state of wellbeing among their workforce, prompting an effective performance [24]. The term toxic or unhealthy organization describes the opposite model, which is defined by a poor organization of work, and also involves high rates of absenteeism and employee turnover [16]. This exposes firms to major financial and productive losses. By contrast, healthy organizations are characterized by a workforce with a high output performance and wellbeing, which in turn leads to robust financial health [25].

According to [26], the organizational climate and the environment in which employees work are psychologically important, having a major impact on good workplace performance and behavior. Hence, there is a positive correlation between organizational health and employee engagement. The link between engagement and organizational health is also related to firm performance, referring to the achievement of its set targets and goals [27].

On the other hand, a work environment designed to support the development of employee engagement—energy, involvement, and effectiveness—will promote the wellbeing and productivity of its employees [28]. From a bidirectional way, wellbeing is important to develop sustainable levels of employee engagement [29].

There is as yet no single and generally accepted definition of the concept of employee engagement. According to [30], “the meaning of the employee engagement concept is unclear”. Even though this engagement has been a hot topic for specialized journals and consultancy firms in recent years, there are still only a handful of studies in the academic literature [31,32]. Highlights in the current state-of-the-art include [33], who defined employee engagement as the workforce’s positive emotional ties and commitment, which entail participation and involvement in their work.

According to [30], an important issue in conceptualizing engagement refers to the approach taken, depending on whether it is a trait (positive outlook on life and work), state (feelings of energy, absorption), or behavior (extra-role behavior). This is why employee engagement has been defined in many different ways [30,34].

An initial schema of employee engagement as cognitive, emotional, and physical resources [35] considers that engaged employees express their authentic self through physical involvement, cognitive awareness, and emotional connections.

Another approach developed by [36] understands engagement as the antithesis of burnout. Along the same lines, the work of [13], based on the Utrecht team, referred to “work engagement” rather than “personal engagement” and proposed that engaged workers are more likely to perform better than their disengaged peers [37].

The satisfaction–engagement approach relates employee engagement to individual involvement and satisfaction, as well as to enthusiasm for work [10]. The development of employee engagement calls for a two-way dialogue between the organization and its workforce. Thus, according to [38], engagement means to be psychologically present when occupying and performing an organizational role. An effective communication process is essential in this management. This research is aligned with [30,39], who contended that job satisfaction is part of employee engagement, although it may reflect a superficial relationship unless it is managed together with all the other core factors involved [10]. In this line of thought, [40] defined employee engagement as the positive attitude of employees towards the organization and its values. With these works, an effort was made to focus on the employee engagement construct.

Finally, [16] identified the ‘multidimensional’ approach to engagement, which is associated with the work of [31], who differentiated between ‘job engagement’ and ‘organizational engagement’, thus suggesting that engagement can have different focuses as with commitment.

In summary, after the analysis of the evolution of engagement as a construct, it can be said that there is a broad consensus among scientists to define employee engagement as a positive, fulfilling, work-related state of mind that is characterized by vigor (energy, resilience, and a strong desire to work hard), dedication (involvement, enthusiasm, pride, and challenge at work), and absorption (concentration and wellbeing during work) [41,42,43]. Rather than a momentary and specific state, engagement refers to a more persistent and pervasive affective–cognitive state that is not focused on any particular object, event, individual, or behavior [13].

Employee engagement refers to the level of positive activation that is felt by workers. More specifically, it involves certain levels of energy, dedication, and concentration at work. When working from home, distractions and the absence of structure may lead to a loss of rhythm and motivation, making us feel anxious because we are not making enough progress [44]. A drastic change in work routines may have an impact on activation, focus, and performance.

Although recent research has investigated engagement as a variable that is subject to day-level variations [45], it seems to reflect a relatively stable phenomenon because of the continued presence of specific job and organizational characteristics [30].

Finally, several studies support a positive effect of employee engagement on wellbeing, [46,47]. In this regard, while engagement is crucial to achieving self-actualization by means of effort and positive affect, wellbeing is the outcome in form of self-fulfillment and self-improvement [48]. Based on the importance of a healthy organization, an in-depth study has now begun of the practices designed not only to prevent risks for employees (physical, psychological, and social) but also to provide an ambit for developing the healthy management of work [49]. Enthusiasm counts for nothing without physical and mental health, so firms and their workforces need to be nurtured.

## 4. A 5Cs Model to Reinforce Employee Engagement and Wellbeing in Times of COVID-19

The great majority of employment initiatives during the pandemic crisis are largely focused on short-time working and temporary unemployment schemes. However, there are also key measures implemented by companies to maintain employment and economic activity, mainly related to occupational health and safety. To do so, many employers also supported wellbeing programs, recognizing the impact caused by the pandemic on workers well- being [50].

Following the changes caused by the COVID-19 health crisis, the world of work is facing numerous challenges and obstacles when handling employee engagement. The lockdown and the social distancing and safety measures introduced to contain the pandemic have an impact on overall wellbeing [51]. The COVID-19 crisis has forced firms to take urgent measures to maintain their business activity and their employees’ safety due to the high level of contagion caused by the global pandemic. It is too early to make a general assessment of whether firms are succeeding in dealing with these changes, as the pandemic is still raging. Yet we can identify certain considerations they should adopt to continue their operations, whether these involve government proposals, current legislation, or examples of successful measures introduced by other companies.

The analysis was based on the five categories proposed as determining factors for reinforcing employee engagement. This study allowed identifying the factors that make up these categories and the metrics that can be used to analyze the workforce’s wellbeing in each organization. Although these indicators can be applied at any stage involving a crisis or time of plenty, they have been used here to reflect how the COVID-19 pandemic is affecting employee wellbeing.

Based on the literature review in this paper, we identified the five factors that influence and reinforce employee engagement [35,52,53,54]: (1) Conciliation: reconciling work and home life, with remote working and flexibility acquiring considerable importance; (2) Cultivation: development schemes for employees; (3) Confidence: through the health and safety of employees, as well as through hands-on leadership; (4) Compensation: rewarding employees’ efforts and covering the additional costs of these difficult times; and (5) Communication: achieving employee participation and engagement.

The conceptualization of the 5C model for reinforcing employee engagement in times of COVID-19 is to be seen as a complementary approach to organizational health and wellbeing, rather than a supplementary one. Different elements of engagement are seen as multiplicative, whereby high scores on one element cannot compensate for low scores on another.

With a view to illustrating the impact this health crisis has had, Table 2 presents the key factors for reinforcing employee engagement and the indicators chosen for their assessment.

### 4.1. Conciliation

According to the study ‘Flexibility at work’ conducted by [55], before the onset of the crisis, 58% of Spanish workers considered that they had everything they needed to do their work from home, and 68.6% were in favor of remote working, although their firm had not implemented it. However, before the pandemic, remote working was a marginal experience, with fewer than 1 in 20 employees in the European Union working in this way regularly in 2018.

During the COVID-19 pandemic, the mass shift to working from home has been one of the key forms of labor market adjustment, thus preventing further job losses. By July 2020, 40% of employees indicated that they were working from home because of the pandemic [50]. However, the figure for the number of employees that can work remotely is lower than the number of households with children or caring for dependent persons (10–20% higher), who cannot be left alone during a lockdown, requiring employees to remain at home.

The plurality of this kind of work renders it difficult to establish a common definition. Nonetheless, [56] drew up an initial classification that identifies the following seven criteria for defining the nature of remote work: location, timeframe, technical, value chain, contractual relationship, and remuneration. Thus, the location criterion corresponded to the physical place where the person is working, as remote working does not necessarily mean working from home. As regards the timeframe, remote work has certain specific characteristics depending on the days of the week, the month, etc. In terms of the working day, and as with any other type of work, it may be full-time or part-time.

Remote working enables a firm to be more efficient because of a more productive workforce using less space, energy, etc. [57]. With most people working from home, pollution levels will be significantly reduced, and sustainability is now a major asset for firms. Those firms that lead the field in the use of this value-added in their favor will have a competitive advantage. Firms have also found that their catchment area for potential new recruits has grown, as “living in the province of the vacant post” will no longer be a requirement for hiring.

For workers, the benefits of remote working derive from the better work-life balance, which improves the level of job satisfaction and engagement [57]. Remote working is essentially viewed as a tool for reconciling work that is based on the intensive use of new technologies, enabling people to work from somewhere other than the firm’s offices or facilities [58]. Reconciling work and family life implies ‘satisfaction and good functioning of work and home, with a minimum of role conflict’ [59]. The aim is to achieve equal opportunities in employment, change traditional stereotypes and roles, and cater for the need to care for and look after dependents [60]. In addition to this ‘win–win’ situation in which both employers and employees gain, there are theories highlighting the difficulties behind this model. Accordingly, it seems that when borders between home and work are intentionally blurred, work pressures spill over into non-work or home life [61].

For the employees, working from home demands more from individuals than what has typically been required to survive in many (though not all) organizational settings because there are fewer scripts for appropriate action, norms for specific behaviors, and patterns for lifelong careers [62]. While their task demands increase as they step outside the protection of an organization, they also often lose a sense of community, stability, and predictability. In the crisis caused by the COVID-19 pandemic, the key factors that concern employees are financial instability, job insecurity, career path uncertainty, work transience, and physical and relational separation.

Yet introducing remote working does not only mean sending the workforce home. Gaining the full potential of this type of work for firms and reinforcing employee engagement call for certain changes, such as establishing a regulatory framework, training for the entire team, setting targets, removing any aspects that might lead to confusion, such as overlapping instructions, breaking processes down to dynamize tasks, and introducing a routine, with a work timetable that has to be upheld to ensure employees do not feel that their private lives are being impinged upon.

Following the [50], during the pandemic, remote workers have been less likely to experience a decline in working hours and more likely to be confident about retaining their job over the post-pandemic period. All this is related to what is called organizational justice or employee’s perceptions of the fairness of treatment received from the firm allowing remote working [63]. Thus, if employees perceive fairness in decision outcomes, they are more likely to feel engaged, and turnover intentions decrease, leading to increases in satisfaction and wellbeing [64]. Thus, it a direct influence on health and quality work-life because it addresses psychological and social risk factors in the workplace as well as organizational climate [65,66]. Thus, the greater the organizational justice, the lower the absenteeism and the better health of employees [67,68]. Accordingly, our first proposition is the following:

**Proposition** **1.** *Conciliation is positively related to employee engagement and wellbeing.*

### 4.2. Cultivation

Within the economic context of COVID-19, not all firms have managed to retain their entire workforce. The need for furlough (referred to as an ERTE in Spain) may affect employee engagement with the firm and lead to greater uncertainty, although the return to their jobs is guaranteed in Spain for six months after the end of an ERTE. Nevertheless, an employee may think that after the ERTE the firm will still need to make redundancies given the prevailing economic climate. Other firms have chosen the path of redundancies, which will have a negative impact when eventually seeking to hire new employees due to the heightened mistrust in the sector as a whole and among the firm’s own employees, as they will question its management. It is vital to try to reduce this uncertainty in order to reinforce engagement.

An individual’s growth and development expectations within the organization, based on their personal characteristics, work experience, skills, and family commitments, have a direct influence on their decision to leave the post [69]. Job resignations are explained by [70] through cusp catastrophe theory, whereby the intention to leave a job is prompted by the work tension that may trigger a psychological state of stress in an employee (referred to as catastrophe), which induces personal and work dissatisfaction (cusp). Furthermore, a dissatisfied employee with signs of stress will show little commitment and proactivity, which will eventually lead to them quitting [71].

Employers need to understand their employee’s expectations and future plans. This has important implications for job designers to ensure that the meaning and purpose of the role are clearly defined. In addition, organizations have to develop cultures in which employees are not discouraged from providing upwards feedback and have honest communication at all levels.

Thus, healthy organizations should provide safe and secure workplaces. This requires employees to be proactively involved in arranging and taking part in organizational practices [72]. These practices include organizational empowerment, which stems from the premise that an organization’s performance and productivity increase when power and control are shared between the firm and its employees [73]. This can lead to lower absenteeism, staff turnover, and resignations [74,75].

According to [76], firms need to consider the employees’ views over how best to engage in wellbeing activities. There is a high correlation between greater opportunities for professional cultivation and development and a lower intention to leave the job. A suitable in-house training plan improves employee attitudes, as well as their expectations and motivation toward their jobs [77]. However, in normal economic circumstances, it is difficult for companies to free up employees for training activities due to higher opportunity costs. During the pandemic period, employee training associated with short-time work and temporary unemployment was also scarce. The main reasons lie in difficulties in predicting the duration of the crisis, limited resources, and lack of planning around training needs, among others [51].

Within today’s business scenario, human resources need to be instructed in the new technologies and tools that firms use. Mastering this new approach to work will provide the skills, motivation, and opportunities for developing and achieving the objectives set for employees. In addition, with a view to maintaining the necessary employee involvement and fostering engagement, coaching is a tool that may help firms to uphold their sustainability and productivity. Coaching is the new ally not only for employees but also for firms in the post-COVID-19 era, inasmuch as it contributes to a return to business and the management of the changes that will arise in the “new normal”. Many of these changes are set to remain, and we will have to learn to live with them, being agile and flexible to stay alert and know how to adapt. Coaching may play its part by prompting people to think and act and by seeking to avoid demotivation, lack of focus, apathy, inaction, etc., favoring the generation of ideas whose realization will improve performance over the medium and long term. On this basis, our second proposition is the following:

**Proposition** **2.** *Cultivation is positively related to employee engagement and wellbeing.*

### 4.3. Confidence

The work context is an important factor affecting people’s health [78]. Although the concept of health is somewhat general, the World Health Organization (WHO) affirms that health is a holistic state of wellbeing, and not simply the absence of illness.

In this regard, organizational injustice may trigger emotional reactions, such as stress [79] or cardiovascular diseases [68]. In addition, this organizational injustice to which an employee may be exposed on a prolonged basis may lead to long-term problems affecting their quality of life, such as sleep disorders and poor-quality rest, inflammation, and a long etcetera [67].

Considering that one of the greatest concerns for employees will be to know whether their workplace is safe. Thus, employee engagement will improve substantially when they are confident that efforts are being made to safeguard their health, strictly complying with official guidelines, and even taking additional measures. According to [80], a “relational-based trust” derives from the quality of the relationship over time than from observation of the other party’s specific behaviors, for example, shared affection or converged interest.

Although firms themselves are responsible for assessing the risk their employees may be exposed to, there are certain general measures they need to guarantee: providing the necessary means to reinforce personal hygiene in the workplace (hand-washing, face coverings, and two-meter social distancing), ensuring the safety of workplaces by cleaning and disinfecting surfaces and equipment, and distributing and organizing spaces onsite (furnishings, corridors) to uphold the two-meter safety distance between employees.

On an organizational level, it is essential to consider the possibility of reassigning tasks and/or remote working, and those establishments that are open to the public need to comply with the measures restricting the number of customers and keeping safety distances, access-control mechanisms, and measures for organizing customers that are queuing outside. Moreover, key steps should be followed for successfully reopening workplaces to ensure the utmost wellbeing for workers and reducing to a minimum the possibility of new infections: (1) quantifying the workforce, (2) assessing the workspace, (3) actively monitoring employees’ biometric parameters in relation to the virus, and (4) prevention and sustainability [81].

Finally, firms need to pay special attention to their employees’ mental health, as they may be exposed to stress, anxiety, or depression. The success of the management of mental health within the context of remote working renders it important to guarantee employees’ privacy. As these expectations are vindicated by management, more powerful degrees of trust may develop. It is derived, for example, from shared affection or converged interest. The overwhelming affection and complete unity of purpose is such that both parties assume a common identity, and each party can represent the other’s interests with their full confidence. These last two are tantamount to what [82] terms “social” trust. Therefore, our third proposition is the following:

**Proposition** **3.** *Confidence is positively related to employee engagement and wellbeing.*

### 4.4. Compensation

Employee compensation or remuneration, normally involving monetary payment, is the way of rewarding a service or job or the person that performs it [83]. It would be a major mistake to remove individual or team productivity bonuses or variable income, demotivating employees in these times of crisis. Wage fairness is a generic concept and does not involve a specific sum of money, as each employee estimates what they consider to be a fair wage depending on their capabilities, experience, and risk. It may not coincide with the wage paid by the firm, which would generate a sense of injustice, being directly related to job satisfaction, engagement, and wellbeing at work [84].

It is very important to plan and implement a target-based incentives policy that responds to the new work scenario. A positive measure involves paying employees a bonus for the risk and possible infection when they enter the workplace, which is most apparent in food companies or retailers. Yet, the management of remote working also involves a series of costs for its proper performance, such as the provision of hardware, accessories for smooth communication with colleagues, and an internet connection. The post-COVID-19 period, with fewer available resources, provides an unbeatable opportunity for firms to apply other types of key benefits that are more financially viable in their compensation plan, such as healthcare insurance.

Non-monetary benefits, such as those of a social nature, are incentives designed to address a specific need and are often provided in a non-cash form. They are less widespread due to the high cost that they incur for the company. In the design of non-monetary benefits, it is important to keep in mind employees’ priorities and lives [85]. As an example, in the current context, a remote worker may start to feel detached and begin building an invisible barrier. This means that the recognition of goals, targets, good results, and involvement in the firm’s daily challenges, for example, has to been seen as a tool that will considerably help to bring a worker closer to an environment that they feel is moving away from them. Accordingly, employee recognition can enhance engagement mainly through public messages in the different media of corporate intercommunication or through a supervisor’s private acknowledgment of their reports, and even extending to the creation of “digital prizes” for team motivation [86].

The post-COVID-19 period, with fewer available resources provides an unbeatable opportunity for firms to apply other types of key benefits that are more financially viable in their compensation plan, such as healthcare insurance. Non-monetary benefits are key factors in the crisis caused by the COVID-19 pandemic. The most useful measures for HR are to provide services and help parents mainly through nursery tickets, a childcare center, and a parenthood charter. These measures are designed to help parents with their day-to-day life, over and above providing financial support. Furthermore, the benefits need to be aligned with HR policies and support the organization’s culture and values. Non-monetary benefits have the greatest impact on employees, given that they will perceive that the company is interested not only in the bottom line but also in each worker’s individual needs outside their job. Accordingly, our fourth proposition is the following:

**Proposition** **4.** *Compensation is positively related to employee engagement and wellbeing.*

### 4.5. Communication

Communication has a key role to play in dealing with the current challenges. In these times of uncertainty, communication should be based on a fluid two-way dialogue. Personal relationships with colleagues, supervisors, and reports are crucial because they are a vital part of the everyday work climate and environment. Affect is one of the factors of interaction among workers that has been revealed through studies and research [87]. Messages should be formulated using empathy and understanding for employee concerns. Acknowledgment is a factor that boosts employee engagement, as a management approach based on mentoring with constant communication, coaching, and a culture of recognition.

Thus, communication is crucial, and even more so now when barriers are being erected between firms and their employees because of the distance involved in remote working. A direct conversation with employees in these times of crisis favors the latter’s engagement. Those employees that have maintained conversations of this nature with their employers or supervisors feel more engaged with the firm, reinforcing their sense of loyalty to their organizations. Feedback may be particularly important in this new world of work, as being outside formal organizations means that workers will be less likely to receive job and career feedback on a consistent, predictable basis, such as during quarterly or annual performance reviews [88].

A good organizational climate fosters open dialogue for greater fluency in communication within the firm. [89] suggested that the notion of supporting others through interpersonal trust might reduce occupational stress and improve employees’ psychological wellbeing and their working life. Furthermore, altruistic behavior favors the development of healthy and trusting personal relationships, also applying to the world of work, where it means fewer conflicts and disagreements [90]. It is essential to avoid disputes in the workplace, as they hinder open communication and learning [91].

Networking is also indispensable, with activities, such as sharing agendas, setting up a website with news on the firm and keeping it updated with each new development that may be of interest to employees and involve the firm’s business, its processes or the sector, as well as the creation of an area for meeting, as a kind of social network where employees may interact.

An area where employees may have their queries answered at a time when there are hardly any routines. In a physical workplace, both formal and informal information flow freely, but unless the management of remote working encourages networking, employees may feel isolated.

More time needs to be dedicated to each employee in order to identify possible demotivation or problem areas. It may be a good time to assess performance, discuss productivity and listen to suggestions, addressing cultural reticence and training needs in technological skills.

This context highlights the digital divide, and it needs to be resolved through training with webinars, for example, to help to address very specific issues or training pills with practical information for performing the job.

**Proposition** **5.** *Communication is positively related to employee engagement and wellbeing.*

Not all these factors can be applied to all employees; these indicators will have a greater bearing on some people than on others, varying according to their personal and professional goals, personality, experiences, or past record. Neither will firms have to deal with the five parameters in the same way. Yet reinforcing employee engagement at this time of uncertainty caused by the pandemic is going to be an essential requirement for retaining talent that remains scarce.

This analysis provides a guideline where firms can find a series of recommendations based on the legal framework, government advice, and successful practices in other companies to manage this pandemic to foster employee engagement and wellbeing.

**Figure 1:**
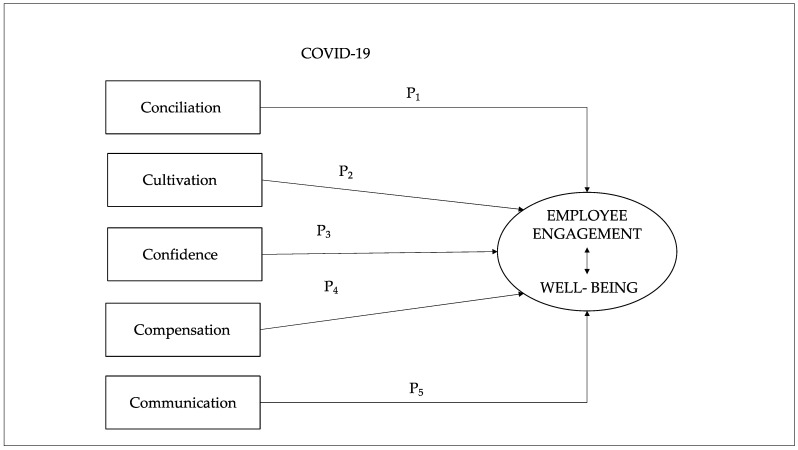
Structural representation of 5C model.

Figure 1 summarizes the structural representation of 5C model and the propositions.

## 5. Conclusions

This article has conducted an in-depth study on how the current pandemic is affecting the management of employee engagement. Based on this analysis, a model has been proposed with the main factors that firms should address to reinforce their employees’ commitment and engagement as they tackle the global turmoil the pandemic has caused. A basic catalog of actions to be adapted and implemented. In this way, each manager will be aware of the main factors of employee engagement that need to be managed and the actions to be carried out for the success of the process. The employee engagement model proposed considers that Conciliation, Cultivation, Confidence, Compensation, and Communication are factors that favor organizational health and wellbeing in the current crisis, and indicators are provided for measuring each one’s achievement.

This research makes two contributions. For the scientific community, the article provides a model of actions for boosting employee engagement, integrating previous theoretical findings reported by specialist authors. The article’s results, therefore, build up a complete and detailed framework, including the most relevant organizational health and wellbeing policies, along with the direct, positive impact we can expect from the crisis caused by the COVID-19 pandemic. This could be the basis for future studies conducted under different external influences (e.g., country or industry) or internal ones (e.g., organizational structure, culture, or leadership style) that may empower or modify the effects of such policies.

Moreover, the study provides a series of practical implications. The health crisis caused by COVID-19 is creating considerable uncertainty among workers, which is compromising their engagement and wellbeing. Given the positive impact of employee engagement and wellbeing on efficiency, productivity, and organizational performance, as well as the current pandemic context, managers should address these factors in order to keep the firm competitive edge. Thus, a roadmap has been drawn up that may serve as a reference for the different managers involved.

Consequently, the results suggest that, in order to achieve employee engagement, managers should focus on facilitating remote working conditions so that employees can reconcile work and family life in this new scenario. Another issue to address is understanding employee’s expectations and future plans and involvement in decision making and organizational practices, in other words, empowerment. Inhouse training is also viewed as an opportunity to improve employee attitudes, expectations, and motivation. In addition, employees will be more engaged when they are confident that efforts are being made to safeguard their health. In this regard, companies should ensure that workplaces comply with all hygienic–sanitary measures, consider reassigning tasks and/or working remotely, and pay special attention to the mental health of their employees. Managers should also realize that a good way to improve engagement is to plan and implement a compensation policy that responds to the new labor scenario, not only with monetary payments but also with non-monetary benefits, which are more financially viable due to the company’s economic circumstances as a result of the pandemic. Finally, firms should facilitate communication, information sharing, and informal relationships among colleagues and supervisors.

The main limitation of this work is the theoretical nature of the study. In order to fill this gap, a futures research line might involve empirically testing the model. Given that the pandemic is still present when the situation stabilizes, the necessary quantitative and qualitative data will be available for undertaking this empirical study. Additionally, it would be interesting to discuss the relationship or differences between employee engagement and motivation.

## Figures and Tables

**Table 1:** Search protocol and results.

Database	WoS
Geographical scope	Global scientific production
Characteristics	Quality indicators: JCR impact factor Immediacy index Times cited Quartile
Documents searched	Topic
Data range	All years to 2021
Search date	6 March 2021
Search terms	“Employee engagement” AND “Wellbeing”
Initial number of documents	148
Inclusion criteria	Article Review
Number of documents	134
Categories filtered process	Management and Business
Number of documents	65
Filtered process	Duplicates Authors not identified Not related to the topic
Final number of documents	59

**Table 2:** 5C model for reinforcing employee engagement in times of COVID-19 and assessment indicators.

Category	Factors	Indicators
1. Conciliation	Remote working Professional-private life Family diversity	Physical and relational separation Productivity Sustainability
2. Cultivation	Professional career New technology Development opportunities	Geographical and communication barriers Furlough (ERTE in Spain) vs. redundancy Coaching
3. Confidence	Health Safety Leadership	Health measures at work Psychological support Employee privacy
4. Compensation	Remuneration Endeavor Non-Monetary benefits	Risk and remote working allowances Services and help for parents Performance incentives
5. Communication	Networking Job and career feedback Involvement	IT resources Two-way dialogue Performance reviews

## Data Availability

The data presented in this study are available on request from the corresponding author.
